# Nonedible Vegetable Oil-Based Polyols in Anticorrosive and Antimicrobial Polyurethane Coatings

**DOI:** 10.3390/polym13183149

**Published:** 2021-09-17

**Authors:** Chandrashekhar K. Patil, Dong Wook Jung, Harishchandra D. Jirimali, Joon Hyun Baik, Vikas V. Gite, Sung Chul Hong

**Affiliations:** 1 HMC, Department of Nanotechnology and Advanced Materials Engineering, Sejong University, Seoul 05006, Korea; chandrashekharpatil999@gmail.com (C.K.P.); jdw6297@naver.com (D.W.J.); 2 Department of Chemistry, Gopal Vidya Nagar, Maliba Campus, Uka Tarsadia University, Bardoli, Surat 394350, Gujrat, India; hdj739@gmail.com; 3 Department of Chemical and Biological Engineering, Sookmyung Women’s University, Seoul 04310, Korea; joonhyun@sookmyung.ac.kr; 4 Department of Polymer Chemistry, School of Chemical Sciences, Kavayitri Bahinabai Chaudhari North Maharashtra University, Jalgaon 425001, Maharashtra, India; vikasgite123@gmail.com

**Keywords:** nonedible vegetable oil, polyol, polyurethane, coating, anticorrosive, antimicrobial

## Abstract

This review describes the preparation of nonedible vegetable oil (NEVO)-based polyols and their application in anticorrosive and antimicrobial polyurethane (PU) coatings. PUs are a class of versatile polymers made up of polyols and isocyanates. Renewable vegetable oils are promising resources for the development of ecofriendly polyols and the corresponding PUs. Researchers are interested in NEVOs because they provide an alternative to critical global food issues. The cultivation of plant resources for NEVOs can also be popularized globally by utilizing marginal land or wastelands. Polyols can be prepared from NEVOs following different conversion routes, including esterification, etherification, amidation, ozonolysis, hydrogenation, hydroformylation, thio-ene, acrylation, and epoxidation. These polyols can be incorporated into the PU network for coating applications. Metal surface corrosion and microbial growth are severe problems that cause enormous economic losses annually. These problems can be overcome by NEVO-based PU coatings, incorporating functional ingredients such as corrosion inhibitors and antimicrobial agents. The preferred coatings have great potential in high performance, smart, and functional applications, including in biomedical fields, to cope with emerging threats such as COVID-19.

## 1. Introduction

The progressive dwindling of fossil fuel resources, coupled with the drastic increase in fossil fuel prices, has sparked feverish activity in the search for alternatives based on renewable resources. Polyols are typically viscous materials that can be converted into value-added polymers. The use of vegetable oils (VOs) in the preparation of polyols has thus gained high attention due to their renewability, low cost, and wide availability. VOs are classified into two general categories, edible and nonedible VO (NEVO). Of the oils, NEVOs have become more attractive for the development of polyols due to their nonfood nature, which is critical for preserving the global food supply. The conversion of NEVOs into polyols can be conducted through various chemical pathways, such as epoxidation, thioene, ozonolysis, hydrogenation, hydroformylation, polyesterification, polyetherification, transesterification, and amidation [1].

**Figure 1:**
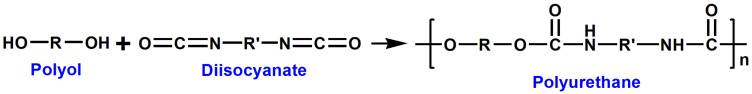
Schematic representation of the preparation of PU from polyol and diisocyanate.

Polyurethane (PU) is a group of versatile polymers invented by Otto Bayer and coworkers in 1937 [2]. As PU is mainly prepared through polyaddition reaction between polyols and multifunctional isocyanates (Figure 1), PU properties can roughly be controlled by the chemical nature and combinations of the polyols and isocyanates, allowing them to be used in various fields including foams (rigid and flexible), adhesives, sealants, and elastomers [2,3]. Due to the presence of urethane groups in their chemical structures, PU particularly possesses excellent chemical resistance, abrasion resistance, thermal stability, and adhesion characteristics, which allow them to be used as coating materials in medical, building, construction, automotive, packaging, marine, furnishing, and electronic fields [4,5].

In this review, different NEVOs and NEVO-based polyols are demonstrated. Preparation of NEVO-based polyols through different chemical pathways are also summarized. The aspects of NEVOs that are covered in this article are their origin, fatty acid composition, physicochemical properties, conversion routes to polyols, and the corresponding PU coatings, including NEVO-based functional PU coatings with anticorrosive and antimicrobial performances. The basics of PUs including diisocyanates and polyols are also introduced.

## 2. Diisocyanates and Polyols for Polyurethanes

### 2.1. Diisocyanates for Polyurethanes

**Figure 2:**
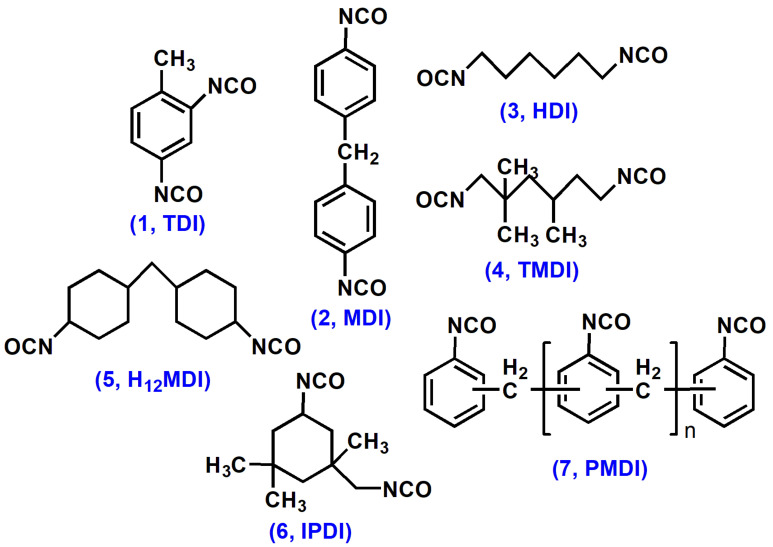
Common aromatic, aliphatic, and cycloaliphatic diisocyanates for PUs.

Diisocyanates can be classified into aromatic, aliphatic, and cyclo-aliphatic (Figure 2). Most common diisocyanates are aromatic diisocyanates such as toluene diisocyanate (1, TDI) and methylene diphenyl diisocyanate (2, MDI). Aliphatic diisocyanates include 1,6-hexamethylene diisocyanate (3, HDI) and 2,2,4-trimethylhexamethylene diisocyanate (4, TMDI), while 4,4′-dicyclohexylmethane diisocyanate (5, H_12_MDI) and isophorone diisocyanate (6, IPDI) are major cycloaliphatic diisocyanates. Polymeric methylene diphenyl diisocyanate (7, PMDI) can also be used. Although aromatic diisocyanates are comparatively less expensive and more reactive than the aliphatic counterpart [6], aromatic diisocyanates often suffer from yellowing and degradation issues under environmental conditions (e.g., UV light). Aliphatic or cycloaliphatic diisocyanates are thus preferred for outdoor applications. The origin of the most of diisocyanates is petroleum resources. However, several reports have claimed that the preparation of diisocyanates is from renewable resources through Curtius rearrangement, including 1,7-heptamethylene diisocyanate and 1,16-diisocyanatohexadec-8-ene from oleic acid [7,8,9].

### 2.2. Conventional Polyols for Polyurethanes

**Figure 3:**
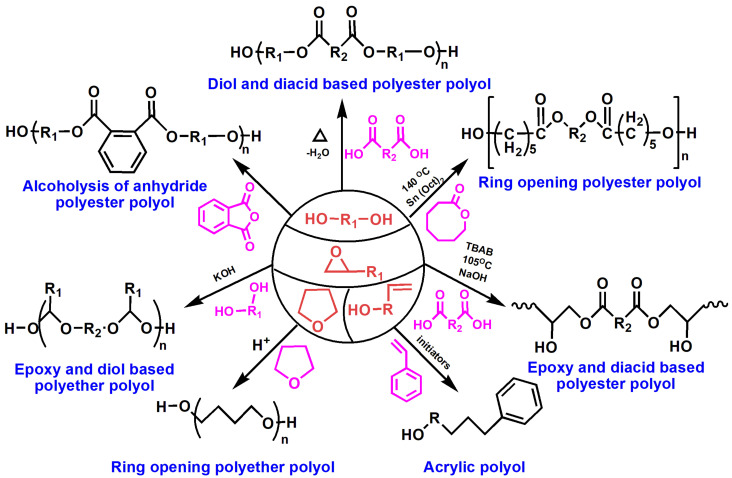
Conventional polyols for PUs from petroleum resources.

Polyols are low-to-moderate molecular weight precursors having two or more hydroxyl groups per molecule. Different types of polyols are commercially available, including polyether, polyester, polycarbonate, polyamides, and acrylic polyols. Most of conventional polyols are obtained from petroleum sources, as exemplified in Figure 3.

Polyester polyols are prepared through different pathways such as acidolysis of epoxides, esterification of acids, and alcoholysis of anhydrides. Polycaprolactone is conventional polyester polyol, which is obtained from ring opening reaction of ε-caprolactone and glycols in the presence of stannous octate as a catalyst. Ring opening oligomerizations of ethylene or propylene oxides afford hydroxyl-terminated polyether polyols. Hyperbanched polyether polyols are also prepared through multibranching polymerizations [10,11,12]. Poly(tetramethylene ether) glycol is also obtained from the ring opening polymerization of tetrahydrofuran. Acrylic polyols are obtained by free radical polymerization of suitable acrylic monomers.

### 2.3. Polyols from Renewable Resources for Polyurethanes

Despite the many advantages, there is a distinct limitation of conventional polyol. Due to diminishing fossil fuels, the rising prices of petroleum based raw materials, and the increase in environmental concerns, academics and industries have been exploring the replacement of petroleum resources with renewable resources. Renewable resources include VOs, cellulose, wood, carbohydrates, starch, and sorbitol [13,14,15,16,17]. Polysaccharides are long chain polymeric carbohydrates formed by monosaccharide units through the glycosidic bond. The examples of polysaccharides are starch, cellulose, and chitosan, etc., which are derived from nature as a structural material to construct cell walls of crustaceans, plants, and other agricultural feed stocks. The polysaccharides have been used for PUs, including cellulose nanocrystal and starch for PU coatings [18,19,20].

**Figure 4:**
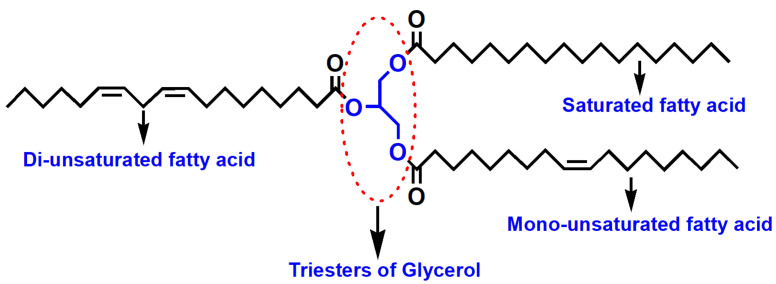
Schematic representation of VOs (triglyceride).

**Figure 5:**
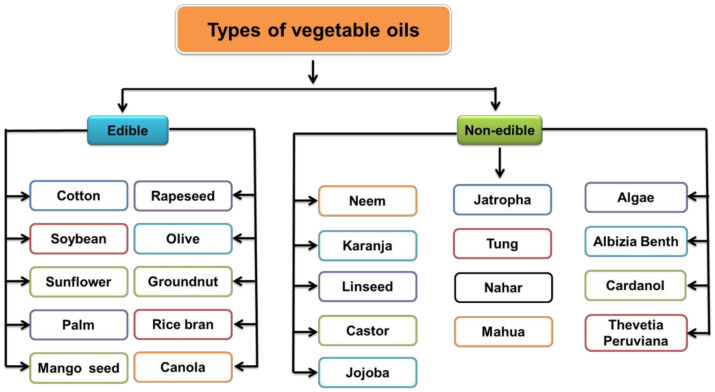
Examples of edible and nonedible VOs.

Among renewable resources, VOs are triglycerides obtained from plants, seeds, and other agricultural feedstocks (Figure 4). Soybean, cotton, palm, sunflower, groundnut, olive, rice bran, cotton seed, and rapeseed oils are falling in the category of edible oils (Figure 5) [21]. The production of edible oils varies depending on the climate and soil conditions, such as palm oil in Southeast Asia, rapeseed oil in Canada, soybean oil in US, coconut oil in Philippines, sunflower oil in Europe, cotton seed oil in India, etc. [22]. However, the rapidly growing world population and extensive human consumption of edible oils may cause significant problems, including starvation in developing countries and higher prices of edible VOs than of fossil fuels.

## 3. Nonedible Vegetable Oils for Polyols

### 3.1. Nonedible Vegetable Oils

The common NEVOs used in the preparation of polyols are listed below. The NEVOs and their fatty acid compositions along with physicochemical properties are presented in Table 1 and Table 2, respectively. Important examples of NEVOs include algae oil (AO), linseed oil (LO), neem seed oil (NO), pongamia glabra seed oils (PO; karanja oil, KO), Nahar seed oil (NAO), castor oil (CO), Albizia benth seed oil (ABO), tung oil (TO), mahua oil (MO), Thevetia peruviana seed oil (TPO), jojoba oil, cashew nutshell oil (cardanol), and jatropha oil (JO) (Figure 5) [23,24,25].

NEVOs can be incorporated in industrial usage due to their low cost; ability to be produced worldwide on marshlands, marginal lands, and wastelands, including under arid/semiarid conditions; and easy cultivation without disturbing food value chains because of toxic ingredients [22]. For example, NO contains azadirachtin, which has an irritating odor, while KO contains flavonoids, such as karanjin, pongamol, tannin and karanjachromene [23]. CO contains ricin, a toxin that may cause fatal gastroenteritis and fever [47]. JO is nonedible because it contains protein curcin and phorbol esters [22]. TPO contains cardiac glycosides, or cardiac toxins, as well as thevetin A, thevetin B, and peruvoside, which have adverse impacts on the heart by inhibiting the sodium-potassium adenosine-triphosphatase enzyme systems [48].

**Figure 6:**
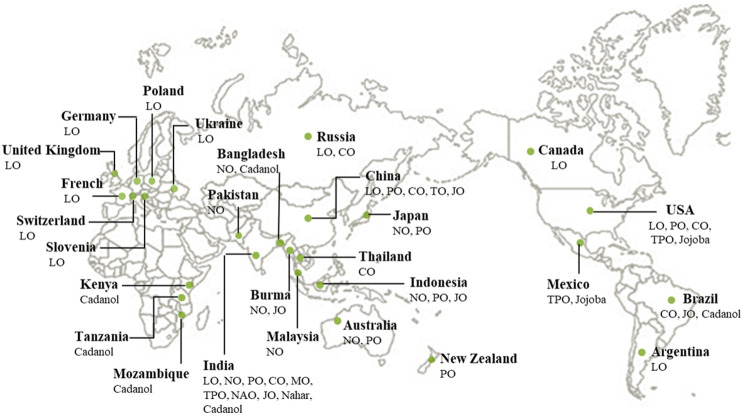
Global growing locations of nonedible oilseed crops.

Approximately 75 nonedible plant species contain more than 30% oils, which can be obtained mainly from their seeds (Figure 6) [22,39,49].

AO is extracted from algae or microalgae and has been a promising candidate for NEVO. The high production possibility of AO is due to the presence of several algae species with high growth rates in many places around the world [50]. Out of 40,000 identified species of algae, microalgae are one of the fastest growing species and oil content of some species exceeds 80% of the dry weight of algae biomass. Oil obtained from microalgae accounts for around 58,700 L/ha [51]. The fatty acid composition of AO shows high percentage of oleic (77.6%) and linoleic (2.2%) acids, followed by linolenic (1.4%), palmitic (9.2%), stearic (3.2%), *cis*-11,14-eicosadienoic acid (1.5%), and other unfamiliar fatty (3.7%) acids (Table 1) [26].

LO is another important NEVO produced largely in Canada, followed by Russia, China, the USA, India, Argentina, and some parts of Europe [37,52,53,54]. LO contains a high percentage of unsaturated fatty acids, viz. linolenic acid (53.2%), followed by oleic (18.5%) and linoleic (17.3%) acids, along with saturated fatty acids, such as palmitic (6.6%) and stearic (4.4%) acids (Table 1). In comparison with other VOs, LO contains a high level of unsaturated acids (α-linolenic), which are responsible for its poor oxidation stability, high rate of polymerization, and drying properties [27]. LO is one of the most popular NEVOs to be used in polymer field. Reports have been published on the development of different types of polyols from LO and their applications in polyester/PU coatings [38,55,56,57,58,59,60,61,62,63,64,65].

Neem trees (*Azadirachta indica A. Juss*) are cultivated in India on arid and dry land. The kernel (seed) of neem trees can be used to extract NO, which contains azadirachtin as a toxic active ingredient. NO is abundantly available in India and the Indian continent, with a production rate of 18,000 per tones in arid land. [28]. NO is also found in several Asian countries, such as Sri Lanka, Pakistan, Bangladesh, Japan, Malaysia, Indonesia and Burma, and in the tropical regions of Australia [37]. NO contains both saturated and unsaturated fatty acids, namely, palmitic (11.9%), stearic (30.0%), arachidic (2.9%), oleic (50.0%), and linoleic (5.2%) acids (Table 1) [28]. NO has many applications in different areas, such as agriculture [66], pharmaceuticals [67], and biodiesel [68,69]. Currently, NO is adopted often in the preparation of functional polymeric coatings.

PO is also known as KO and is extracted from karanja seeds. Approximately 110,000 tons of seeds are collected every year in India, especially in the eastern states of India and other states, such as Andhra Pradesh, Karnataka, Tamil Nadu, Assam, and Western Ghats [70]. PO is also found in China, Japan, Australia, New Zealand, and the USA [29,30]. PO possesses unsaturated and saturated fatty acids; in particular, it contains oleic acid (44.5~71.3%) at higher percentages than linoleic (10.8~18.3%), stearic (2.4~8.9%), palmitic (3.7~7.9%), behenic (4.2~5.3%), arachidic (2.2~4.7%), and lignoceric (1.1~3.5%) acids [29,30]. Crude PO has also been used to produce biodiesel by the methyl ester route for substitution of petroleum-based fuels [71,72].

Nahar (*Mesua ferrea* L.) is an oil seed plant in that is widely grown in the northeastern region of India and contains a high oil percentage (70~75%). The fatty acid composition of NAO comprises primarily oleic (52.3%) and linoleic (22.3%) acids, followed by low levels of palmitic (15.9%) and stearic (9.5%) acids [31]. Although many polyesters or epoxy resins from NAO have been reported [39,73,74], only a few examples can be found for PU resin with NAO [75,76,77,78].

CO is unique among all VOs due to the presence of hydroxyl groups on ricinoleic acid (92~95%) [32]. CO is cheap, widely available, and in high demand due to its inherent hydroxyl groups [32]. The main countries that produce CO are India, China, Brazil, Thailand, and Russia. Castor is grown on about 1.26 × 10^6^ ha area with about 1.14 × 10^6^ tons production and world average productivity is about 0.90 ton/ha [79].

ABO contains linoleic (47.9%) and oleic (20.7%) acids as major fatty acids, followed by palmitic (14.1%), stearic (11.2%), and linolenic (5.8%) fatty acids [33]. TO is also known as Chinese wood oil and is extracted from tung tree seeds. TO contains α-eleostearic acid (77.0~82.0%) as a major fatty acid, followed by oleic (3.5~12.7%) and linoleic (8.0~10.0%) acids [34]. TO has fast drying properties and a fast oxidative polymerization capability in the presence of oxygen, which can be catalyzed by cobalt [80,81]. In addition to its use in coating formulations, TO is also used in the preparation of PU composites, emulsions, and foams [82].

MO is extracted from mahua seeds (*Madhuca indica*). MO is found abundantly in tropical regions of India, Madhya Pradesh, Andhra Pradesh, Bihar, Gujarat, Uttar Pradesh, and Odisha. The main fatty acid composition of MO is oleic (35.8%), stearic (22.5%), palmitic (22.3%), and linoleic (16.6%) fatty acids [35].

*Thevetia peruviana* (yellow oleander) is an ornamental plant belonging to the *Apocynaceae* family and is found on the American, Asian and African continents and in regions of India, specifically in Assam [83]. TPO contains both saturated (36.4%) and unsaturated (63.6%) fatty acids, including oleic (43.7%), palmitic (23.3%), linoleic (19.9%), stearic (10.7%), and arachidic (2.4%) acids [36,83]. Only a limited report is available on the utilization of TPO in polymer preparation.

Jojoba is native to the California deserts, Mexico, and Arizona [84]. The jojoba tree is from the Simmondsiaceae family, and the seed contains approximately 40~50 wt.% oil with a fatty acid composition of oleic acid (43.5~66%) and linoleic acid (25.2~34.4%) [37].

Cashew nutshell liquid (also called cashew oil, cashew nutshell oil) is a natural alkenyl phenolic compound obtained from cashew nutshells as an agricultural byproduct. Cashew nutshell liquid has a low cost, is abundantly available (450,000 metric tons per year), and contains linear unsaturated phenolic derivatives, viz. cardanol, 2-methyl-cardol, anacardic acid, and cardol [85]. The production of cashew nutshell liquid has increased with the increase in cashew nut tree (*Anacardium occidentale* L.) plantations in Brazil and other parts of the world, such as India, Bangladesh, Kenya, Tanzania, Mozambique, tropical regions of Africa, and Southeast/Far-East Asia [85,86]. It contains 18~27% phenolic moiety that has unsaturated 15-carbon chain [87,88]. Cashew nutshell liquid has been used in many industrially important chemicals because of its unique structure and desirable properties, such as hydrophobicity, flexibility, weatherability, and acid and alkali resistances [89]. The reactive phenolic hydroxyl group and an unsaturated C15 alkyl chain make cashew nutshell liquid a good platform for chemical modifications in the form of polyols that can be converted into coatings, foams, and paints [90,91,92,93,94].

Jatropha is known to have 170 different species, of which 66 species have been identified as major sources of oil and are cultivated in Central/South America, Africa, India, and Southeast Asia, covering a global area of approximately 900,000 ha [95]. More than 85 percent of jatropha plantations are in Asia, chiefly Myanmar, India, China, and Indonesia. Africa accounts for around 12 percent or approximately 120,000 ha, while Latin America has approximately 20,000 ha of jatropha, mostly in Brazil [95]. JO contains mainly unsaturated fatty acid, such as oleic (34.3~44.7%), linoleic (31.4~43.2%), palmitic (13.6~15.1%), and stearic acids (7.1~7.4%) [37].

### 3.2. Chemical Transformation of Nonedible Vegetable Oils to Polyols

**Figure 7:**
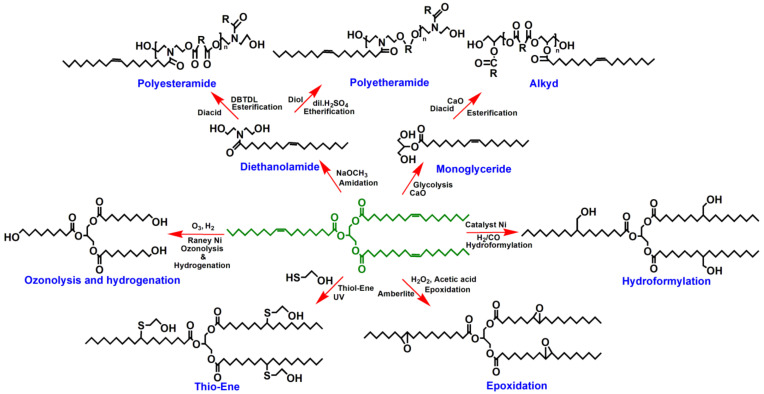
Schematic representation of the preparation of different polyols from VOs.

Polyols can be prepared from NEVOs by esterification, etherification, amidation, epoxidation, etc., taking advantage of its functional groups in their structures (Figure 4). The preparation of polyols from NEVOs is quite similar to that from edible VOs, following several pathways as schematically demonstrated in Figure 7.

One of the most prominent routes is through polyesteramide (PEsA), which can be prepared by esterification of VO-based diethanolamides with dicarboxylic acids or anhydrides [96]. Prior to the preparation of PEsAs, diethanolamides are prepared by the amidation of VOs by reacting them with diethanolamine in the presence of a suitable catalyst [96,97]. Polyetheramide (PEtA) is another class of polyols that first requires the preparation of diethanolamides, which are further reacted with diols to be converted into PEtAs [50]. Alkyds are the oldest polymeric resins prepared with VOs and contain ester moieties in their polymeric chains [16].

Polyols with terminal primary hydroxyl groups can be prepared by the ozonolysis route by treating VOs with ozone to form ozonide and further reduction with Raney nickel [97]. Polyols can also be prepared by the hydroformylation process of VOs with the addition of hydroxymethyl groups to the carbon-carbon double bonds of the fatty acid chains. VO reacts with a mixture of CO and H_2_ in the presence of a catalyst to give aldehydes and is further reduced with H_2_ to obtain hydroxyl groups [97]. In the hydroformylation process, the most frequently used catalysts are cobalt carbonyl complexes selectively modified by tertiary phosphate ligands and tertiary phosphine rhodium carbonyl species [13].

Epoxidation of VOs is one of the most popular routes for the preparation of polyols and can be achieved by different processes, such as epoxidation by peracids, hydrogen peroxide, dioxirane, phase transfer catalysts, and chemoenzymatic species [16,21,72,97,98]. Generally, epoxidation is carried out at the carbon-carbon double bonds of fatty acids using a peracid with hydrogen peroxide and acetic/formic acid, followed by opening of the oxirane ring with proton donors.

VO-based polyols can also be prepared by a single-step thiol-ene reaction in VOs with thiol compounds under ultraviolet light for the conversion of double bonds into polyols [45,99,100,101,102]. Acrylation, vinylation, metallation, and other chemical pathways can also be employed for the preparation of polyols to produce better dried, glossy, scratch-resistant, impact-resistant, flexible, and corrosion-resistant coatings. The presence of hydroxyl, ester, oxirane, amide, carbonyl, acrylic, carboxyl, and urethane groups may provide good adhesion characteristics of the corresponding coatings to substrates.

## 4. Nonedible Vegetable Oil-Based Polyurethane Coatings

Corrosion and microbial growth on metal surfaces are severe problems that cause enormous economic losses annually in many sectors, including in marine, medical, packaging, construction, transportation, oil refining, pipelines, and petrochemical industries. Corrosion is damaging to industrial machines, power plants, ships, bridges, water treatment plants, etc. Trillions of dollars are spent on maintenance every year. Antiviral coatings on personal protective equipment are currently in high demand for vaccines and other medical applications due to COVID-19. Biofouling of marine coatings is another important issue pertaining to microbial growth that results in tremendous economic losses (approximately $30~50 billion every year) [26]. Table 3 summarizes the synthetic routes for PU coatings from different NEVOs. The various ingredients used to afford anticorrosion and antibacterial functionalities to PU coatings are also summarized.

### 4.1. Nonedible Vegetable Oil-Based Anticorrosive Polyurethane Coatings

#### 4.1.1. Anticorrosive Polyurethane Coatings: Adhesion

Corrosion is considered a metallic cancer. It is a natural phenomenon causing deterioration of metal through chemical/electrochemical reaction with environment. Corrosion cannot be completely eradicated, but can be overcome in a number of ways, such as cleaning the environment, alloying metals, using inhibitors, using paints and coatings, etc. Among these methods, the use of polymeric coatings is widely accepted since they are easier to apply and cheaper than other methods. These provide protection against environmental attack on metals for quite a long time [114,155].

**Figure 8:**
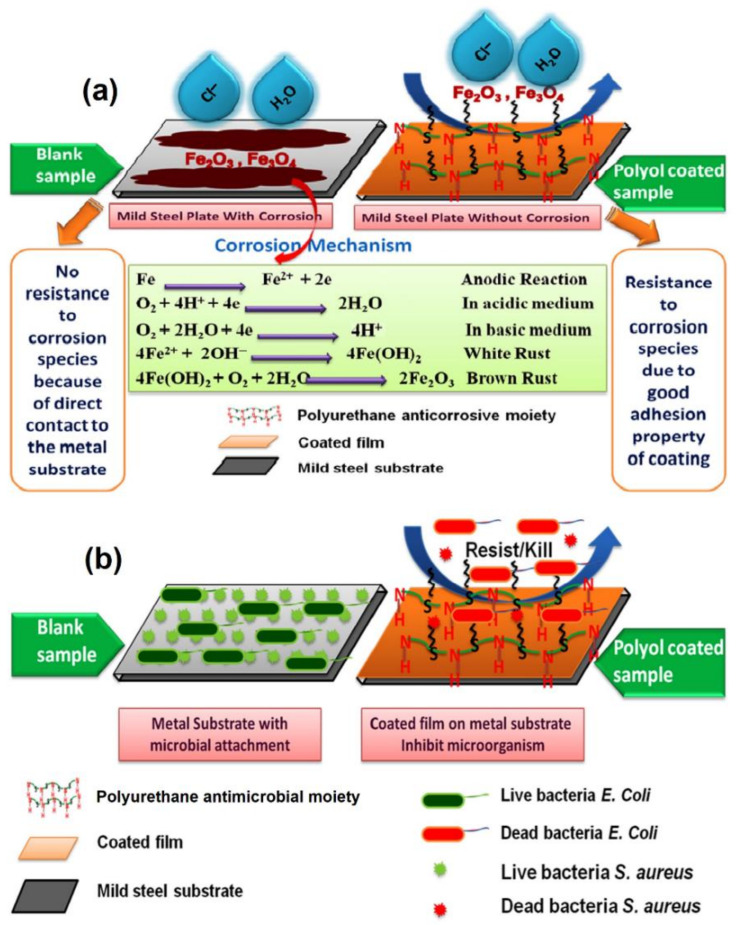
Proposed mechanisms of the anticorrosive (**a**) and antimicrobial (**b**) performances of PU coatings. This image is reproduced from reference [103] with permission. Copyright of the Reactive and Functional Polymers (Elsevier).

Corrosion processes on metallic surfaces involve conversion of metal atoms into an ionic state through oxidation reaction. Due to the electro-neutrality principle, electrons released from the metal atom need to be taken up by some oxidizing agent. Water contains dissolved atmospheric oxygen, which readily serves the purpose of electron acceptor and is electrochemically reduced to hydroxyl ions. The metal ions and hydroxyl ions combine together to produce metal hydroxide, which further reacts with more oxygen to form hydrated metal oxide (i.e., corrosion, Figure 8a) [120].

Therefore, prohibiting the permeation of corrosive media is a basic requirement for anticorrosive performances. Uniform and well-adhered PU coating over the metal substrate due to the presence of heteroatoms in its chemical structure, such as oxygen and nitrogen in urethane linkages, thus make it an inherently excellent anticorrosive coating material. The many lone pairs of electrons on the heteroatoms interact with the vacant d-orbital of metal to form coordination bonds [63,156]. The protection mechanism is simply through the barrier action attributed to the well-adhered PU coating [120].

**Figure 9:**
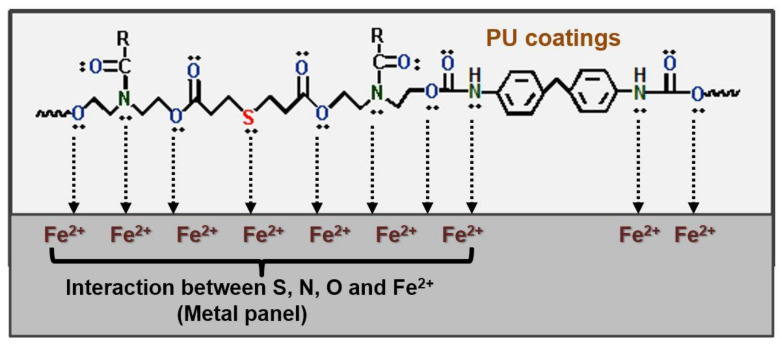
Possible interactions of heteroatoms of PU coatings with Fe (metal substrate).

For an even stronger affinity with the metal surface, researchers have tried to incorporate heterocyclic or aliphatic heterocompounds with additional heteroatoms, such as nitrogen, sulfur, and oxygen atoms, into PUs. The proposed interaction mechanism of the heteroatoms with a metal substrate is demonstrated in Figure 9. Nitrogen, sulfur, and oxygen atoms contain a lone pair of electrons that can bond with metal substrates to form coordination bridges. Such strong adhesion does not allow corrosive ions to penetrate the metal surface [63,120].

**Figure 10:**
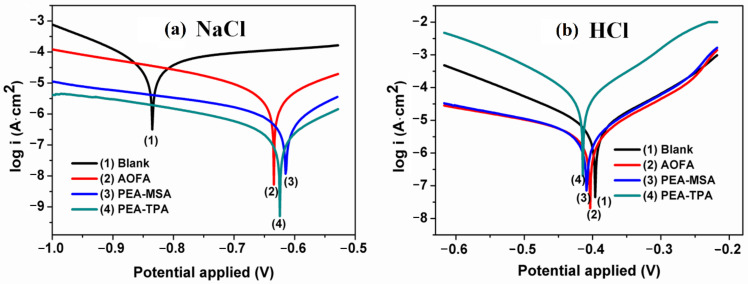
Tafel plots of electrochemical corrosion studied by PDP of uncoated (blank) and PU coated MS panels in 3.5% NaCl (**a**) and 0.5 M HCl (**b**) solutions. This image is reproduced from reference [103] with permission. Copyright of the Reactive and Functional Polymers (Elsevier).

Corrosion inhibitors containing sulfur atoms, such as mercaptosuccinic acid and thiodipropionic acid, were used for the development of anticorrosive PU coatings from AO. The AO-based coatings showed a good barrier property against NaCl and HCl corrosive media. The anticorrosion activity of these coatings is mainly attributed to the excellent adhesion and strong interactions between the sulfur of mercaptosuccinic acid and thiodipropionic acid with a metal substrate [103]. The projected anticorrosion reaction mechanism and Tafel plots are shown in Figure 8 and Figure 10, respectively. Researchers also utilized bis(4-aminophenyl)sulfone [139,140], pyridine [63], piperazine [120], and 1-thioglycerol/orthophosphoric acid [157] as corrosion inhibitors.

LO-based cyclocarbonate precursors for nonisocyanate PU coatings were reported by Pouladi et al., through the epoxidation and carbonation of LO, followed by a reaction with diethylenetriamine [109]. CO-based nonisocyanate PU coating was also reported by Sabnis et al. [143]. Hydroxyl groups produced by the reaction between cyclocarbonate and amines provided excellent adhesion to the substrate.

PU coatings based on alkyd resins of NO were prepared by the monoglyceride route, and they exhibited good flexibility and high gloss due to the high content of oil and long fatty acid chain of the prepared polyol [121]. KO-based polyols were also prepared through epoxidation and esterification, in which KO-based PU coatings exhibited good thermal and physicomechanical properties [23].

Fatty amide is another candidate that provides not only improved physicochemical properties but also better anticorrosive performances of PUs. Fatty amide contains an additional nitrogen atom in its chemical structure, improving its adhesion and anticorrosive characteristics. Poly(urethane fatty amide) (PUFA) showed better coating performance in terms of gloss, bending, chemical resistance, impact resistance, scratch resistance, and thermal resistance. Ahmad et al., reported LO-based PUFA composite with conducting polymer, poly(1-naphthylamine) [113]. The dense and continuous structure of the tested coatings was consistent with their ability to protect the metal substrate from corrosion. The PUFA matrix functioned as a barrier coating and prevented the penetration of aggressive ions, while the conducting polymer reacted with the substrate to form a passive oxide layer [113]. NO fatty amide-based PU coatings showed good anticorrosive performance due to the excellent adhesion of the coatings with a metal substrate [119]. The LO-based one-pack PUFA resin was reported by Ahmad et al., including amidation of LO with diethanolamine followed by a reaction with TDI [65]. Gite et al. reported NO-based PU coatings using a trimer of isophorone diisocyanate and fatty amide polyol [119]. Karak et al. reported poly(urethane amide) coating resins from purified NAO and TDI [75]. General coating performances of the resins were better than those of corresponding polyester or PEsA coatings, exhibiting good chemical resistance in various test media.

PEsA polyols have been developed by the reaction of NEVO fatty amides with diacids to improve the properties of coatings, such as corrosion resistance, thermal properties, and physicomechanical properties [62]. Gite et al. prepared AO-based PEsA polyols using different diacids and utilized them for the development of anticorrosive and antimicrobial PU coatings [26,103,104]. LO-based PEsA polyols have been developed by the reaction of LO fatty amides with ethylenediaminetetraacetic acid (EDTA) [62]. PU coatings prepared from pyridine dicarboxylic acid-LO PEsA polyols showed good corrosion resistance [63]. PEsA polyols have been developed by the reaction of LO, PO, and CO-based fatty amides with itaconic acids to improve the properties of coatings [111]. The PEsA-based PU coatings have fatty amide groups and polar urethanes linkages, forming H bonds with the metal substrates and enhancing their adhesion performance.

Alam et al. designed an anticorrosive PEsA polyol by replacing typical dicarboxylic acids with EDTA [62], ethylene glycol tetraacetic acid [124], and itaconic acid/*trans*-1,2-diaminocyclohexane-N,N,N′,N′,-tetraacetic acid [46,154], employing LO, PO, and JO, respectively. The PU coatings prepared from EDTA and *trans*-1,2-diaminocyclohexane-N,N,N′,N′-tetraacetic acid-based polyols have a combination of amine groups, ester groups, urethane groups, and pendant alkyl chains. JO-derived poly(esteramide urethane)/fumed silica nanocomposite PU coatings were demonstrated by Alam et al., which showed good adhesion, hardness, gloss, and anticorrosive properties [154]. NO-based PEsA polyols were also prepared for self-healing anticorrosive PU coatings, where polyamidoamine-based polyurea microcapsules were filled with LO [28].

Despite their many advantages, PEsA coatings possess some drawbacks, such as poor alkali resistance and low thermal stability. Therefore, to achieve good performance, researchers have moved toward a new class of intermediates, such as PEtA, which exhibits good mechanical and chemical properties.

PU prepared from AO-based PEtA polyols showed good anticorrosive capability due to the nitrogen containing polyols with long aliphatic chain of AO [50]. Alam et al. reported the use of LO for the preparation of PEtA resins using bisphenol-A and resorcinol, affording improved mechanical properties, such as scratch hardness, impact resistance, and bending resistance [64]. Shaik et al. also reported the use of LO for the preparation of PEtA resins in the presence of Fe_2_O_3_ NPs as a corrosion inhibitor [112]. Further example of PU coatings with additional heteroatoms includes a polyol developed from PO and ethane-1,2-di(azomethine) bisphenol (hydroxyl terminated Schiff base) [125]. The polyol showed improved anticorrosive properties due to the presence of imine groups.

Akintayo et al. prepared PEtA polyol-based coatings from ABO and bisphenol-A with different loadings of TDI [144]. The prepared coatings achieved good flexibility, adhesion, scratch hardness properties, and impact resistance with increased loading of TDI (10–20% TDI loading). These observed results may be due to the presence of polar urethane, amide, ether groups and fatty acid chains, which form bonds with the substrate [144]. A MO-modified PEtA polyol-based PU was reported and exhibited good anticorrosion performance against acid and salt solutions [35]. Yemul et al. converted MO into PEtA polyol using bisphenol-A, which was reacted with methylene diphenyl diisocyanate/TDI and resulted in good coating properties [35]. JO-based PEtA for PU coatings were presented by Alam et al. [153].

#### 4.1.2. Anticorrosive Polyurethane Coatings: Crosslinking

A review of various PUs elucidated the influence of the crosslinking on the properties of the PUs due to the formation of the three-dimensional network. The crosslinked structure reduces the void space, pores, and free volume in the coating structure, making the path for the penetration of the corrosive electrolyte more tortuous [123]. Ahmad et al. delivered a pioneering work on PU coatings prepared from LO by epoxidation and hydroxylation, showing good anticorrosive properties due to their highly crosslinked structure [105]. Gite et al. compared the anticorrosive properties of NO-based alkyd-modified PU coatings by using an immersion method, exhibiting better anticorrosive nature due to increased crosslinking reaction [121]. Ranade et al. explored the integration of PO for better crosslinking of PU coating, inhibiting electrolyte mass flow through the coating [123]. Gite et al. showed that the combination of CO monoglycerides and IPDI trimer forms better crosslinked PUs, exhibiting good resistance towards acids, alkalis and solvents [129]. Siyanbola et al., introduced poly(epichlorohydrin-triol) crosslinker to CO-based PU coatings. The improved crosslinking and adhesion on mild steel gave a better chemical resistance [132]. Recently, Benedetti et al. claimed improved corrosion protection of aluminum alloy AA2024 by tannin-modified CO-based PU coating, which favor crosslinking due to a high number of -OH groups in the chemical structure [136,137].

Yang et al. demonstrated the Diels–Alder reaction of TO and acrylates for potential ultraviolet-curable nonisocyanate PU coatings [145]. Epoxy resin was synthesized from cardanol oil and further utilized for the preparation of PU coatings [93,151]. The corrosion resistance showed improvement in barrier properties by oxidative crosslinking [93]. Sabnis et al., also prepared PU coatings by the reaction of cadanol-based diglycidyl ether with various multifunctional acids [152]. The crosslinking densities of cured coatings were observed to have great impact on chemical resistance, thermal stability and anticorrosive performance of prepared PU coatings.

Hyperbranched PU is not a crosslinked polymer network, but it has received considerable attention due to its unusual chain architectures and characteristics, such as high solubility, low hydrodynamic diameter, low melting temperature and solution viscosity [158]. Hyperbranched urethane alkyd coatings were prepared from LO and isocyanate trimer by Naik et al., showing excellent mechanical and corrosion resistance properties [116]. Karak et al. reported that the monoglyceride CO is an effective intermediate for the preparation of hyperbranched PU, exhibiting good resistance under various chemical environments due to the presence of high hydrophobic part [32].

#### 4.1.3. Anticorrosive Polyurethane Coatings: Organic-Inorganic Hybrids

The combinations of organic with inorganic compounds have been studied to fulfill the necessities of PU coatings with specific properties such as anticorrosive performances. The corrosion protection mechanism of inorganic filler can be explained based on the formation of impermeable barrier phase at the interface of corrosive media and the metal substrate, which prohibits the permeation of electrolyte ions through coatings [114].

The siloxane/urethane hybrid exhibits good anticorrosive performances. PU coatings were prepared from epoxidized CO with different proportions of metallic inorganic/organic agents such as titanium isopropoxide with 3-aminopropyltriethoxysilane or tetraethoxyorthosilicate (TEOS), exhibiting improved anticorrosion/barrier characteristics due to Si–O–Si linkages, which provides excellent adhesion and hydrophobic property to the hybrid PU films [159]. Similarly, silica containing hybrid PUFA coatings were prepared through the reaction of LO fatty amide and TEOS [114]. The corrosion resistance of the PU coatings was increased with increasing loading of TEOS, again due to the presence of O–Si–O hydrophobic groups. The coating prevented the penetrations of corrosive ions of alkaline NaOH medium, whereas it failed to protect the substrate against acidic medium because of the breakdown of amide bond of PUFAS [114]. Shaik et al. prepared a hybrid PU by acrylated alkoxy silane polyol through radical copolymerization, which is synthesized from 3-(trimethoxysilyl)propyl methacrylate and CO [128]. Raju et al. reported silica (3-aminopropyltrimethoxysilane)/ZnO hybrid PU coatings from TPO [149].

Alumina can also be incorporated into PU framework to enhance physicomechanical and anticorrosion characteristics [110]. The performances of alumina-filled PEsA urethane coatings were much better than those of corresponding PEsA urethane coatings. Benedetti et al. also prepared PU coatings with inorganic corrosion inhibitor, zinc flake, using polyester polyol and the CO-based prepolymer on the aluminum alloy [135].

Protective and reinforced one coating systems for steel were prepared from PU and red iron oxide pigments as an anticorrosive filler by Morsi et al. [133]. CO-based PU acted as a binder in the formulation with red iron oxide pigments, where the long aliphatic chains of CO played an important role in higher protection activity.

Of late, boron has been adopted in the backbone of polymers to develop semi-inorganic polymers [134,160]. Anticorrosive performance of PU coatings containing boron with LO, PO, and TO-based polyols were also reported [98,107,126]. In these formulations, boron played the roles of crosslinker and modifier, which increased number of –O–B–O– linkage to improve the anticorrosion characteristics [107].

#### 4.1.4. Anticorrosive Polyurethane Coatings with Additives: Nanocomposites

In recent decades, researchers have successfully synthesized polymeric resins with nanoparticles (NPs) for various applications. These resins with NPs have shown good coating characteristics and thermal stabilities. Nanocomposites are thus an emerging class of materials used to develop coatings, which may offer significant barrier properties for corrosion protection. In this context, incorporation of NPs in NEVO-based PU has been a potential way to improve the anticorrosion performances of the coatings [27,161]. These coatings exhibited enhanced anticorrosion performances by blocking the vulnerable sites of steel panels with the nanofillers.

**Figure 11:**
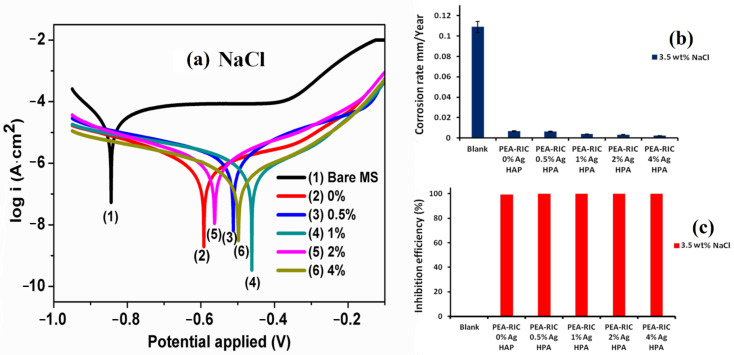
Tafel plots (**a**), corrosion rate (**b**), and inhibition efficiency (**c**) of PU coatings tested in a 3.5 wt% NaCl solution with or without Ag-HAP NPs. This image was reproduced from reference [104] with permission. Copyright of the Progress in Organic Coatings (Elsevier).

Various nanofillers, such as fumed silica, TiO_2_, ZnO, and Fe_2_O_3_ NPs, have been reported in NEVO-based polyols and PUs [112,118,149,150,154]. PU with silver (Ag)-doped hydroxyapatite (HAP) NPs and the PEsA polyol were prepared from AO and ricinoleic acid, exhibiting anticorrosion characteristics (Table 4, Figure 11) [104]. The improved anticorrosion characteristics were attributed to the nonporous, hydrophobic nature of the coating and the presence of hydrolytically stable urethane bonds, which provided better adhesion of the coatings to the substrate.

Carbon nanofillers can be another option for anticorrosive PU coatings. Graphene oxide (GOx) nanofillers can be an example of the carbon nanofillers [122]. The prepared coatings restricted the formation of rust and bacterial growth on the metal surface and formed a barrier to aggressive species (O_2_, H_2_O, and Cl^−^). The results were attributed to the π-π interactions of GOx and double bonds of fatty acid chains [122]. Siyanbola et al., developed CO-based PU with carbon nanorods [127]. The carboxylated carbon nanomaterials were synthesized from incinerated *Eucalyptus globulus* leaves, affording PU composites with improved hydrophobicity and anticorrosion properties.

#### 4.1.5. Anticorrosive Polyurethane Coatings with Additives: Microcapsules

The preparation of smart anticorrosive PUs using renewable and ecofriendly resources has been a trendy research topic in recent years. One of the most versatile ways to prepare smart polymers is to use microcapsule-containing active ingredients, e.g., self-healing agents and corrosion inhibitors [162,163,164].

Microcapsules containing LO have autoxidative properties due to its drying characteristics, which repair minor cracks without manual intervention [165,166,167]. Self-healing anticorrosive PU coatings of LO-based microcapsules have been reported [28]. The coatings exhibited good anticorrosion performances in electrolyte solution due to the healing properties of LO [28].

**Figure 12:**
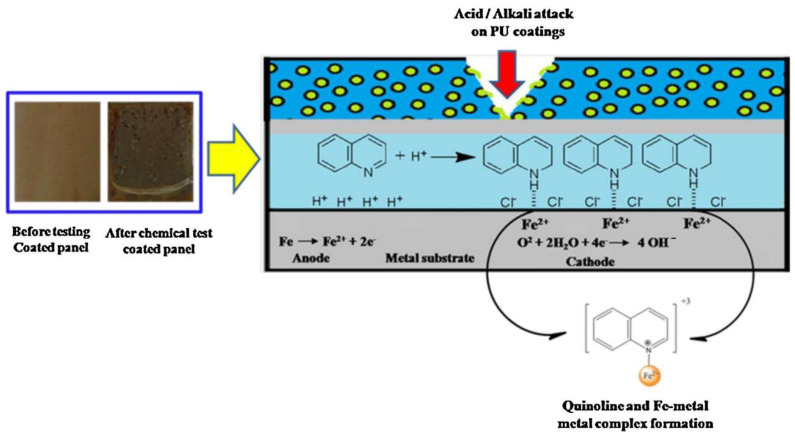
Possible corrosion inhibition and complex formation mechanism of encapsulated quinoline-loaded NAPP-MDI coatings on Fe-metal. This image is reproduced from reference [117] with permission. Copyright of the Industrial Crops and Products (Elsevier).

Gite et al. reported the synthesis of acetylated polyester polyols from NO and converted them into anticorrosive PU coatings with the incorporation of encapsulated quinoline microcapsules as a corrosion inhibitor [117]. The coatings slowly released a quinoline inhibitor from the microcapsules, protecting the substrate for an extended period (Figure 12) [117].

### 4.2. Nonedible Vegetable Oil-Based Antimicrobial Polyurethane Coatings

The global concerns about the risk of bacterial infection have been growing over the past decades [168]. In this regard, coatings with antibacterial activity have offered an approach to limit the spread of bacterial infections in many areas such as medical devices, healthcare products, hospitals/dental office equipment, and food/drug manufacturing [168,169].

The long hydrocarbon chains of fatty triester residues containing hydroxyl groups, carbamate groups, and isocyanate groups can elicit an inhibitory response. These active agents damage lipid complexes in cell membranes or dehydrate bacterial cells. The PUs prepared from the CO-based epoxy- and LO-based hydroxy-terminated polyol have cytocompatibility and antibacterial activity against *S. aureus* and *E. coli*, which allow them to be used in wound dressing applications [106,115,170]. CO has good compatibility with polycaprolactone and improved the antibacterial performance of PU coatings [138]. Antibacterial PU coatings produced from epoxidized LO and PO-based polyol were also tested against *E. coli* [106], and the bioflavonoids karanjin and pongamol played an important role in the performance of the coatings [171]. MO-based PUFA also exhibited good antibacterial activities [146,147,148].

Different agents can be incorporated into the formulation of PU coatings to provide better antibacterial activity [131]. Quaternary ammonium salts showed broad spectrum antibacterial activity against both Gram-positive and Gram-negative bacteria and a high inhibition rate over a wide range of pH values. PU containing covalently bonded quaternary ammonium salts showed good antibacterial performance [131].

Metallohybrid PU composite coatings were developed from copper acetate and LO-based polyol [108,172], which showed notable antibacterial performance against both *E. coli* and *S. aureus*. Parent oil, copper acetate, and CuO bound to lipids in the bacterial cell wall, which disrupted the integrity of the cytoplasmic membrane and blocked the entry of essential nutrients into cells.

Antimicrobial PU coatings were also prepared from Zn- and Cd-incorporated LO-based poly(esteramide-urethane), exhibiting superior performance to previously reported systems [57,172,173]. Moreover, PU nanocomposite coatings obtained from TPO polyols and siloxane-modified ZnO showed antibacterial performance towards *E. coli* and *S. aureus* [149]. Self-healable CO-based hyperbranched PU coatings modified with sulfur NPs and reduced GOx showed good microbial inhibition against both bacteria (*S. aureus* and *E. coli*) and fungi (*C. albicans*) [174], as evidenced by the membrane disruption and irregularity of the cellular structures.

**Figure 13:**
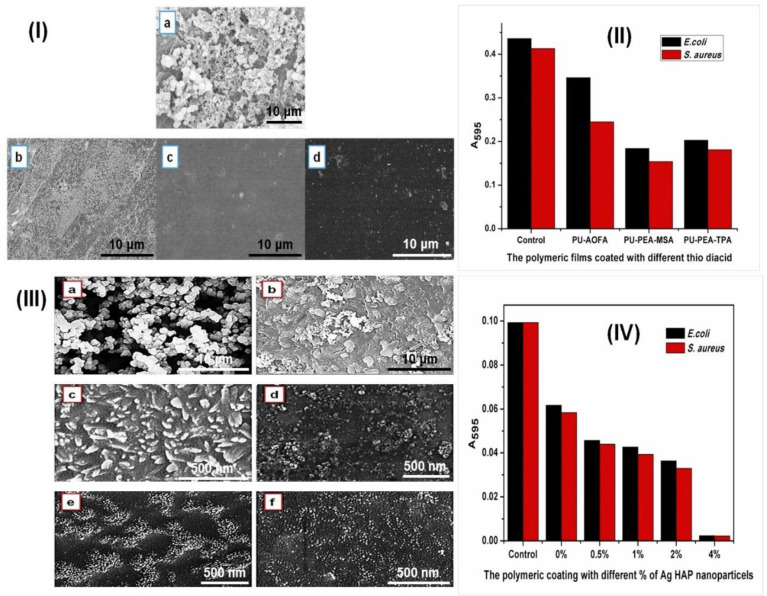
SEM images of PU coatings tested against *E. coli* and *S. aureus* containing PEsA- mercaptosuccinic acid/PEsA-thiodipropionic acid (**I**; a, uncoated metal tray; b, PU coating with algae oil fatty amide; c, PU coating with PEsA-mercaptosuccinic acid; d, PU coating with PEsA-thiodipropionic acid) and Ag-HAP NPs (**III**; a, MS panel; b, PU coating without Ag-HAP; c, PU coating with 0.5% Ag HAP NPs; d, 1% Ag HAP NPs; e, 2% Ag HAP NPs; f, 4% Ag HAP NPs). Graphical representations of uncoated and coated MS panels tested against *E. coli* and *S. aureus* containing mercaptosuccinic acid/thiodipropionic acid (**II**) and Ag-HPA NPs (**IV**): Absorbance at 595 nm as a measure of cell growth. These images are reproduced from references [103,104] with permission. Copyright of the Reactive and Functional Polymers and Progress in Organic Coatings (Elsevier).

Ag has long been known for its good antimicrobial efficiency and hence has been utilized in many polymer composites. HAP NPs are another example that can be prepared from biowaste materials, i.e., waste egg shells. Gite et al. developed antibacterial PU coatings by employing PEsA, PEtA, Ag-doped eggshell HAP, and thio-diacids [26,50,103,104]. The prepared PU coatings showed good antibacterial potential against Gram-positive and Gram-negative bacteria due to the presence of a high percentage of oleic acid in AO, which can inhibit bacterial growth [175]. The observations of ruptured surfaces and decreased bacterial growth on PU-coated surfaces were confirmed by SEM analysis, as shown in Figure 13. The penetration effect of Ag NPs into the surface of the cell membrane can disturb the permeability and respiration functions of the cell [176,177]. CO-based waterborne antibacterial PU nanocomposites were prepared by incorporating Ag-halloysite composites, catalyzing the production of oxygen radicals to inhibit the growth of microorganisms [178].

TiO_2_ NPs exhibited good antimicrobial activity and strongly bonded to electron donor groups in biological molecules containing oxygen, nitrogen, and sulfur, which destroyed the outermost layer of microorganisms [141]. TiO_2_ incorporated CO-based PU nanocomposites and waterborne PU dispersions showed good antibacterial and antifungal activities against *S. aureus, E. coli*, *B. pasteurii*, *Vibrio parahaemolyticus*, and other fungal species [179].

Hybrid PU coatings with CO/LO-based PU with TEOS exhibited good antibacterial behavior against *E. coli* and *S. aureus* due to the large surface area of silica NPs [142]. A PU hybrid containing 3-amino propyl trimethoxy silane and 3-glycidoxy propyl trimethoxy silane showed mild biocidal inhibition properties against bacteria *E. coli* and *S. aureus* due to the synergetic effect of hydroxyl, urethane, and Si components in the prepared PU film, which are responsible for breaking down bacterial proteins and restricting the cellular activity [130].

## 5. Conclusions

The most popular renewable precursor for the synthesis of biopolyols is vegetable oil. The selection of raw materials plays a substantial role in the preparation of polyols for the development of ecofriendly polyurethanes. Although several factors affect the selection process, such as productivity, viability, cost, environmental effects, conversion efficiency, reusability, and the presence of modification sites, nonedible vegetable oils are an excellent platform due to their nonedible nature. Several chemical pathways have been demonstrated to convert nonedible vegetable oils into polyols. The mechanical, chemical, thermal, anticorrosive, and antimicrobial characteristics of the nonedible vegetable oil-based polyols can be improved by advanced chemical modification strategies. New nonedible vegetable oils with additional functionalities and active ingredients can be good options for further applications of these materials, as demonstrated by the incorporation of nanofillers, silica, silver, hydroxyapatite, and inorganic metalloids. The use of microcapsules, triazines, thiocompounds, hybrids, and inhibitors has also been highlighted in this review. The preferred coatings have great potential in the field of high-performance, smart, and functional applications, including biomedical fields.

## Figures and Tables

**Table 1:** Fatty acid composition of NEVOs.

NEVOs	Type of Fatty Acids	Oleic	Linoleic	Linol-Enic	Palmitic	Stearic	Ricino-Leic	α-Eleos-Tearic Acid	cis-11,14-Eicosa-Dienoic Acid	Arac-Hidic	Ligno-Ceric	Behenic	Unknown	References
	No. of Carbon	18	18	18	16	18	18	18	20	20	24	22	-	
	No. of C=C Bonds	1	2	3	0	0	1	3	2	0	0	0	-	
AO		77.6	2.2	1.4	9.2	3.2	-	-	1.5	-	-	-	3.7	[26]
LO		18.5	17.3	53.2	6.6	4.4	-	-	-	-	-	-	-	[27]
NO		50.0	5.2	-	11.9	30.0	-	-	-	2.9	-	-	-	[28]
PO (KO)		44.5~71.3	10.8~18.3	-	3.7~7.9	2.4~8.9	-	-	-	2.2~4.7	1.1~3.5	4.2~5.3	-	[29,30]
NAO		52.3	22.3	-	15.9	9.5	-	-	-	-	-	-	-	[31]
CO		-	-	-	-	-	92.0~95.0	-	-	-	-	-	5.0~8.0	[32]
ABO		20.7	47.9	5.8	14.1	11.2								[33]
TO		3.5~12.7	8.0~10.0	-	-	-	-	77.0~82.0		-	-	-	-	[34]
MO		35.8	16.6	-	22.3	22.5	-	-	-	-	-	-	2.8	[35]
TPO		43.7	19.9	-	23.3	10.7	-	-	-	2.4	-	-	-	[36]
Jojoba		43.5~66	25.2~34.4											[37]
JO		34.3~44.7	31.4~43.2		13.6~15.1	7.1~7.4								[37]

**Table 2:** Physicochemical characteristics of common NEVOs.

NEVOs	Specific Gravity (g/cm^3^) (25 °C)	Refractive Index	Saponi-Fication Value (mg KOH/g)	Iodine Value (g of I_2_/100 g)	Acid Value (mg KOH/g)	Hydroxyl Value (mg KOH/g)	References
AO	N.A. ^a^	N.A. ^a^	193	85	0.2	-	[26]
LO	0.896	1.478	160	181	8.3	-	[38]
NO	0.920	1.503	186	65	1.0	-	[28]
PO (KO)	0.938	1.467	182	89.9	11.5	-	[30]
NAO	0.890	1.473	260	89.3	14.3	-	[30,31,39]
CO	0.913	1.474	185	82	0.6	165	[40,41]
ABO	0.915	1.473	185	104.5	8.1	0.3	[33]
TO	0.915	1.517	187	167	2.6	-	[34,42]
MO	0.904	1.477	187	62.4	0.4		[35]
TPO	0.912	1.464	121	71.4	0.66	-	[30]
Jojoba	0.866	1.464	100	95	2.0	-	[43,44]
Cardanol	0.941~0.924	1693~1686	47~58	215~235	12.1~15.4	170~250	[45]
JO	0.903	1.475	190	97	2.1	-	[40,46]

^a^ Not available.

**Table 3:** Synthetic intermediate (or pathway) and active ingredients of anticorrosive and antibacterial PU coatings.

NEVOs	Synthetic Intermediate/Pathway	Functional Ingredients	Functionality	References
AO	PEsA	Mercaptosuccinic acid, thiodipropionic acid	Antibacterial, anticorrosive	[103]
	PEsA, alkyd	Phthalic anhydride, itaconic, maleic, dimer acids	Antibacterial, anticorrosive	[26]
	PEsA	Ricinoleic acid, silver (Ag)-hydroxyapatite (HAP) nanoparticles (NPs)	Antibacterial, anticorrosive	[104]
	PEtA	Fatty amide	Antibacterial, anticorrosive	[50]
LO	Epoxidation, hydroxylation	LO, TDI	Anticorrosive	[105]
	Epoxidation, hydroxylation	Long hydrocarbon chains of fatty triester residues	Antibacterial	[106]
	Epoxidation, hydroxylation, polyesterification	Boric acid	Anticorrosive	[107]
	Epoxidation, esterification	Copper acetate	Antibacterial	[108]
	Epoxidation, carbonation	Hydroxyl groups of nonisocyate PU	Anticorrosive	[109]
	PEsA	Fatty amide, pyridine	Anticorrosive	[63]
	PEsA	Fatty amide, EDTA	Anticorrosive	[62]
	PEsA	Fatty amide, aluminium hydroxide	Anticorrosive	[110]
	PEsA	Fatty amide	Anticorrosive	[111]
	PEtA	Fatty amide, resorcinol	Anticorrosive	[64]
	PEtA	Fatty amide, Fe_2_O_3_ NPs	Anticorrosive	[112]
	Fatty amide	Fatty amide, poly(1-naphthylamine)	Anticorrosive	[113]
	Diethanolamine	Fatty amide	Anticorrosive	[65]
	Diethanolamide	Fatty amide, tetraethoxyorthosilicate (TEOS)	Anticorrosive	[114]
	Polyesterification	-	Antibacterial	[115]
	Alkyd	Hyperbranching structure	Anticorrosive	[116]
NO	Epoxidation	Quinoline microcapsule	Anticorrosive	[117]
	PEtA	Fatty amide, TiO_2_ NP	Anticorrosive	[118]
	Fatty amide	Diethanolamine	Anticorrosive	[119]
	Fatty amide	Fatty amide, piperazine	Anticorrosive	[120]
	Alkyd	Anhydride, itaconic, and dimer fatty acids	Anticorrosive	[121]
PO (KO)	Epoxidation, hydroxylation	Bioflavonoids-karanjin and pongamol	Antibacterial	[106]
	Epoxidation, acrylation	Acrylic acid, graphene oxide (GOx)	Antibacterial, anticorrosive	[122]
	Monoglyceride, polyol mixture	-	Anticorrosive	[123]
	PEsA	Fatty amide, ethylene glycol tetraacetic acid	Anticorrosive	[124]
	PEsA	Fatty amide	Anticorrosive	[111]
	PEtA	Fatty amide, ethane 1,2-di(azomethine) Bisphenol	Anticorrosive	[125]
	PEsA, boronation	Fatty amide, boric acid	Anticorrosive	[126]
NAO	Polyol mixture	Fatty amide	Anticorrosive	[75]
CO	-	COOH functionalized carbon nanorods	Anticorrosive	[127]
	Acrylation	Alkoxy silane	Anticorrosive	[128]
	Monoglyceride	-	Anticorrosive	[32]
	Monoglyceride	Trimer of isocyanate	Anticorrosive	[129]
	Polyol mixture	3-Amino propyl trimethoxy silane, 3-glycidoxy propyl trimethoxy silane	Antibacterial	[130]
	Polyol mixture	Benzyl triethanol ammonium chloride	Antibacterial	[131]
	Polyol mixture	Poly(epichlorohydrin)-triol, PMDI	Antibacterial, anticorrosive	[132]
	Polyol mixture	Red iron oxide pigment	anticorrosive	[133]
	Esterification	Boric acid, phthalic anhydride	Anticorrosive	[134]
	Transesterification	Zinc flakes	Anticorrosive	[135]
	Transesterification	Tannin	Anticorrosive	[136,137]
	Transesterification	-	Anticorrosive	[40]
	Transesterification, polyol mixture	Pentaerythritol, polycaprolactone,	Antibacterial	[138]
	Transesterification, copolymerization	Bis(4-aminophenyl)sulfone	Anticorrosive	[139,140]
	PEsA	Fatty amide, TiO_2_	Anticorrosive, antibacterial	[141]
	PEsA	Fatty amide	Anticorrosive	[111]
	Hydrolysis, condensation	TEOS	Anticorrosive, antibacterial	[142]
	Epoxidation	Tris-2-hydroxy ethyl isocyanurate, hydroxyl groups	Anticorrosive	[143]
ABO	PEtA	Fatty amide	Anticorrosive	[144]
TO	Hydroxylation, alcoholysis	Anhydrides, boric acid	Anticorrosive	[98]
	Diels-Alder	Glycidyl methacrylate	Anticorrosive	[145]
MO	Fatty amide	Fatty amide, diethanolamine	Anticorrosive, Antibacterial	[146]
	PEtA	Fatty amide	Anticorrosive	[35]
	PEtA	Fatty amide, diglycidyl ether of bisphenol-A	Antibacterial	[147]
	PEtA	Fatty amide, Bisphenol-C	Antibacterial	[148]
TPO	PEsA, glycerides	Fatty amide, ZnO NPs	Anticorrosive	[149,150]
Cardanol	Epoxidation, hydroxylation	-	Anticorrosive	[151]
	Epoxidation, hydroxylation	-	Anticorrosive	[152]
	Cardanol-epoxy condensation	Autoxidative catalyst drier	Anticorrosive	[93]
JO	PEtA	Fatty amide, hydroquinone	Anticorrosive	[153]
	PEsA	Fatty amide, itaconic acid	Anticorrosive	[46]
	PEsA	Fatty amide, fumed silica NPs	Anticorrosive	[154]

**Table 4:** Electrochemical corrosion study of uncoated (blank), PU-coated mild steel (0% Ag-HAP) and PU with Ag-HAP NPs in a 3.5 wt% NaCl corrosive solution. This table is reproduced from reference [104] with permission. Copyright of the Progress in Organic Coatings (Elsevier).

Ag-HAP Content	E_corr_ (mV)	I_corr_ (µA cm^−2^)	R_p_ (kΩ)	Corrosion Rate (mm/Year)	Inhibition Efficiency (%)
Blank	−842.90	9.38130	1.7244	0.10901	−
0%	−600.450	0.0056990	57.0990	0.00662	99.39
0.5%	−563.080	0.0053659	38.3980	0.00623	99.94
1%	−459.450	0.0032487	81.4370	0.00377	99.96
2%	−591.100	0.0027605	85.4140	0.00320	99.97
4%	−517.430	0.0018696	128.700	0.00217	99.98

## Abbreviations

ABO	Albizia benth seed oil
Ag	silver
AO	algae oil
CO	castor oil
EDTA	ethylenediaminetetraacetic acid
GOx	graphene oxide
HAP	hydroxyapatite
JO	jatropha oil
KO	karanja oil
LO	linseed oil
MO	mahua oil
NAO	Nahar seed oil
NEVO	nonedible vegetable oil
NO	neem seed oil
NP	nanoparticle
PEsA	polyesteramide
PEtA	polyetheramide
PO	pongamia glabra seed oils
PU	polyurethane
PUFA	poly(urethane fatty amide)
TDI	toluene diisocyanate
TEOS	tetraethoxyorthosilicate
TO	tung oil
TPO	thevetia peruviana seed oil
VO	vegetable oil
